# Improving Access to Antimicrobial Prescribing Guidelines in 4 African Countries: Development and Pilot Implementation of an App and Cross-Sectional Assessment of Attitudes and Behaviour Survey of Healthcare Workers and Patients

**DOI:** 10.3390/antibiotics9090555

**Published:** 2020-08-29

**Authors:** Omotayo Olaoye, Chloe Tuck, Wei Ping Khor, Roisin McMenamin, Luke Hudson, Mike Northall, Edwin Panford-Quainoo, Derrick Mawuena Asima, Diane Ashiru-Oredope

**Affiliations:** 1 Commonwealth Pharmacists Association, London E1W 1AW, UK; omotayo.olaoye@commonwealthpharmacy.org (O.O.); chloe.tuck@commonwealthpharmacy.org (C.T.); weiping.khor@commonwealthpharmacy.org (W.P.K.); R.Mcmenamin@uea.ac.uk (R.M.); 2 Horizon Strategic Partners, London EC3R 8HL, UK; luke@horizonsp.co.uk (L.H.); mike@horizonsp.co.uk (M.N.); 3 Liverpool School of Tropical Medicine, University of Liverpool, Liverpool L3 5QA, UK; 248964@lstmed.ac.uk; 4 LEKMA Hospital, Teshie, Accra P.O. BOX MS 216, Ghana; mawuenaasima@gmail.com

**Keywords:** CwPAMS App, smartphone apps, antimicrobial prescribing, pharmacy

## Abstract

Smartphone apps have proven to be an effective and acceptable resource for accessing information on antimicrobial prescribing. The purpose of the study is to highlight the development and implementation of a smartphone/mobile app (app) for antimicrobial prescribing guidelines (the Commonwealth Partnerships for Antimicrobial Stewardship—CwPAMS App) in Ghana, Tanzania, Uganda and Zambia and to evaluate patients’ and healthcare providers’ perspectives on the use of the App in one of the participating institutions. Two structured cross-sectional questionnaires containing Likert scale, multiple-choice, and open-ended questions were issued to patients and healthcare workers six months after the introduction of the app at one of the hospital sites. Metrics of the use of the app for a one-year period were also obtained. Download and use of the app peaked between September and November 2019 with pharmacists accounting for the profession that the most frequently accessed the app. More than half of the responding patients had a positive attitude to the use of the app by health professionals. Results also revealed that more than 80% of health care workers who had used the CwPAMS App were comfortable using a smartphone/mobile device on a ward round, considered the app very useful, and found it to improve their awareness of antimicrobial stewardship, including documentation of the indication and duration for antimicrobials on the drug chart. It also encouraged pharmacists and nurses to challenge inappropriate antimicrobial prescribing. Overall, our findings suggest that its use as a guide to antimicrobial prescribing sparked positive responses from patients and health professionals. Further studies will be useful in identifying the long-term consequences of the use of the CwPAMS App and scope to implement in other settings, in order to guide future innovations and wider use.

## 1. Introduction

Antimicrobial stewardship programs in hospitals are focused on optimising antimicrobial prescribing to improve individual patient care, decrease healthcare costs and combat antimicrobial resistance [1]. The availability of accurate and up-to-date information is important to guide the right diagnosis and prescription of antimicrobials. Healthcare providers’ attempts to access this information are influenced by previous training, availability of the information, ability to access and leverage technology [2]. There has been a recent rise in the use of smartphones generally across global population and it is predicted to be rising fastest in Africa. There has been increased development of smartphone apps designed for use in healthcare, including in the area of antimicrobial stewardship [3,4,5,6,7]. Current research in medicine has shown that the use of mobile phones and devices in medical settings is more popular and is increasingly being brought to the fore of international research [1]. For instance, recent studies have shown that 52% of smartphone users access medical information through their devices [8]. A study by Kamerow, Chief scientist and Associate editor for the British Medical Journal, revealed that there are approximately 100,000 health-oriented smartphone apps and, by the year 2015, over 500 million smartphone owners worldwide will use these apps [9]. The study also highlighted that, although designed for health professionals, around 15% of health apps are now marketed to patients to help them monitor, evaluate, and transmit medical data such as blood pressure and body weight among other health checks [9]. The author also stated that the use of these apps was higher amongst the younger population, females, and people who earned a higher income. Similarly, results from a longitudinal study of 206 medical doctors working at Hannover Medical School, Germany in the summer of 2012 and spring of 2014 also revealed a rapid increase in the use of mobile devices in medical settings during patient interaction and professional collaboration [10]. This significant increase was observed in both the frequency of use and the expansion of the areas of application of these devices. Smartphones have specific features that support their increasing use in healthcare delivery and behavioural interventions. They are highly portable, more convenient, cost-effective and interconnected compared to reference books and computers, thus promoting improved communication and the sharing of knowledge, data and resources among health professionals and as well as facilitating regular updates as new data becomes available [11,12,13]. Furthermore, the ability of smartphones to use internal sensors to deduce context including emotions, location and activity has greatly increased their relevance in the consistent monitoring and tracking of health-related behaviours and healthcare delivery [14,15,16,17,18,19]. In the early days of their use, there was a significant paucity of academic research on users’ viewpoints and experiences with the use of these apps. The recent literature has provided positive feedback on the acceptability and workability of smartphone apps although it has also been recognized that this evolving technology may raise concerns regarding privacy and security [17,20]. In the past decade, there has been a rapid increase in the use of mobile phones in Africa [18]. There has also been a rapid integration of mobile health technologies and telecommunication into the healthcare system, especially in low and middle-income countries. In addition to this there has been an increased investment in mobile healthcare interventions including the use of these technologies for behavioural change communication [19]. With the increasing burden of communicable and non-communicable diseases in Africa, low-cost mobile health technology has the potential to make healthcare more accessible to disadvantaged communities [21]. For example, in Zambia and Ghana adverse event reporting apps were developed by medical regulatory authorities in 2019 [22]. The Zambia Medicines Regulatory Authority—ZAMRA also launched Adverse Drug Reaction Application (ADRA), a new mobile application for android phone users for reporting adverse medicines reactions in 2017 [23]. Furthermore, apps have been used to identify falsified and substandard medicines in Kenya [24]. These technologies also offer great solutions aimed at improving the speed, safety and quality of healthcare provision in resource-constrained settings by providing easy access to local and international guidelines and resources. The purpose of the study is to highlight the development and implementation of an app to support prudent antimicrobial prescribing and improved antimicrobial stewardship practice; as part of the Commonwealth Partnerships for Antimicrobial Stewardship (CwPAMS) programme in Ghana, Tanzania, Uganda and Zambia and to conduct a pilot study assessing patients and healthcare providers’ perspectives on the use of the app in one of the hospitals in Ghana.

## 2. Results

### 2.1. App Metrics 1 Year from Launch (April 2019–May 2020)

The Commonwealth Partnerships for Antimicrobial Stewardship App was developed to improve antimicrobial prescribing and stewardship practices among health professionals in Ghana, Tanzania, Uganda and Zambia. The app provides, for the first time in the four countries, easy access to infection management resources to improve appropriate use of antimicrobials in line with national and international guidelines. Following the launch of the app in four countries, there were 530 downloads of the app and 2,795 guide opens within 12 months. Ghana had more page hits (50.3%) than Uganda (31%), Tanzania (13%), Zambia (1.9%) and others (3.8%) (Table 1). The most visited section of the app was the National Prescribing Guidelines, accounting for 66.1% of the total number of page hits while the section for Updates on antimicrobial resistance (AMR) (coming soon) was the least visited (0.7%). Pharmacists (51.1%) and nurses (20.4%) accounted for the highest number of registered users while pharmacists (64.1%) and medical doctors (20.3%) had the highest frequency of downloads and guide opens (Table 2).

### 2.2. Cross-Sectional Survey Studies

A cross-sectional attitude and behaviour survey was carried out on patients and healthcare professionals to determine their attitudes/views on the use of antimicrobial prescribing guidelines by health professionals. A total of 47 patients and 38 health professionals participated in the survey; response rates were 51% and 38%, respectively.

#### 2.2.1. Demographics

Demographics presented in Table 3 shows that respondents comprise various age groups and educational qualifications and professions.

#### 2.2.2. Patients

##### Patients’ Responses to the Use of Smartphone Mobile Apps in Healthcare Delivery

Patients’ views on the use of the app by health professionals obtained using a Likert scale of five options (Strongly agree, Agree, Neutral, Disagree and Strongly disagree) are presented in Table 4. More than 50% of patients had a positive attitude to the use of smartphone apps by health professionals and the fact that it increases the quality of healthcare offered by health professionals and quickens access to healthcare. Patients’ greatest concern was that the use of smart phone mobile apps in healthcare delivery could be a distraction to healthcare provision. This was followed by concerns that their data may not be protected/secure and that mobile devices may not be technically reliable enough. Patients’ least concern was that the health professional “may not be competent enough”.

##### Patients’ Concerns with Their Health Professionals’ Use of Smartphone Apps by Age and Education

The highest proportion of patients who had no concerns with their use of smartphone apps by health professionals were aged 26–35 (71.4%). This was followed by patients aged 68 and above (66.7%), 18–25 (64.0%), 56–67 (60.0%) and 46–55 (33.3%) in descending order. Patients aged 36–45 had concerns with health professionals’ use of smartphone apps. Patients with the most concern with health professionals’ use of smartphones were aged 46-55. With respect to patients’ highest level of education, patients with tertiary education (63.2% had the least concern with health professionals’ use of smartphone apps while patients with basic primary education (25%) had the most concern.

##### Patients’ Preferences for Health Professionals’ Use to Access Medicines Information

The highest proportion of patients wanted health professionals to use a computer or laptop (38.3%). This was followed by smartphone mobile apps (23.4%), reference books (6.4%) and tablets (6.4%) in descending order. A computer/laptop/reference book was preferred by 6.4% of patients while 2.1% preferred any of a smartphone, computer/laptop or tablet, a smartphone, computer/laptop or reference book, a smartphone or tablet, and a computer/laptop, reference book or tablet. Additionally, 10.6% of patients had no preference (n = 47).

#### 2.2.3. Healthcare Workers

##### Use of the CwPAMS App and Other Sources of Information

Thirty-eight healthcare workers (HCWs) comprising of four doctors, eighteen nurses, six pharmacists and ten other healthcare workers participated in the survey. On a daily basis, mobile phones (28.9%) and printed posters (13.2%) were most predominantly used by the HCWs, while tablets and computers (7.9% each) were the least used devices (Table 5). Mobile phones were used more than once a day by 60.5% of healthcare workers. Percentages of healthcare workers who had never used a tablet, pocketbook, printed posters and computers were 47.4%, 28.9%, 26.3% and 21.0%, respectively. Healthcare workers’ responses showed that many respondents had not consulted the CwPAMS App for antimicrobial prescribing information. The British National Formulary (BNF)/National guidelines, a printed copy of standard treatment guidelines, senior colleagues and junior doctors were mostly consulted daily. In descending order, internet search engines, senior colleagues and pharmacists were consulted more than once a day. No additional source of information on antimicrobial prescribing was mentioned.

##### Use of the CwPAMS App and Other Sources of Information on Antimicrobial Prescribing by Profession

**Figure 1:**
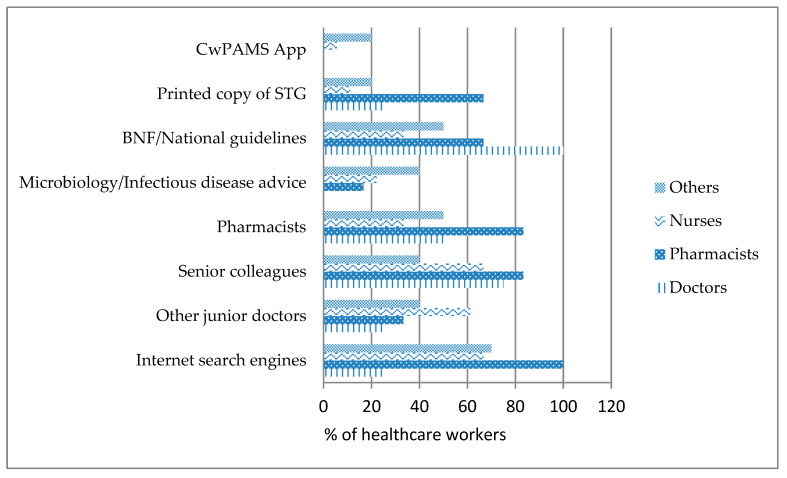
Use of the CwPAMS App and other sources of information on Antimicrobial prescribing by profession.

An assessment of the various sources of information on antimicrobial prescribing used by healthcare workers showed that the CwPAMS App was mostly used by nurses and other health workers. BNF and National guidelines were mostly used by doctors (100%) and pharmacists (66.7%) and least used by nurses (33.3%). Internet search engines were mostly used by pharmacists (100%) and least used by doctors (25%) (See Figure 1). Pharmacists were seen to refer to their senior colleagues for antibiotic information more than doctors, nurses and other health professionals. More doctors and other healthcare workers (midwives, dispensing technicians and medication counter assistants) sought information from pharmacists than nurses. Printed copies of the standard treatment guidelines were mostly used by pharmacists and least used by nurses.

##### Assessment of Standard Treatment Guidelines and Drug Resistant Infections

All responding healthcare practitioners admitted being concerned about the emergence of drug resistant infections while 79.0% agreed or strongly agreed that these guidelines are easy to access. A total of 44.7% stated that they preferred their senior’s preferences over standard treatment guidelines. Only 18.5% preferred to use non-standard treatment guidelines for antimicrobial prescribing while 13.2% felt the standard treatment guidelines did not apply to their patients (Table 6).

##### Perception and Assessment of the CwPAMS Smartphone App

All healthcare workers who had used the App agreed that the app was very useful, relevant to their patient population and considered it the best way to access standard antimicrobial treatment guidelines. In addition, they all felt comfortable using a smartphone on a ward round, admitting that the app increased their awareness of antimicrobial stewardship and encouraged them to challenge inappropriate prescribing and to document the indication and duration for antimicrobials on the drug chart. Furthermore, participants found the country-specific standard treatment guidelines most useful. This was followed by the WHO Essential Medicines list section and the Antimicrobial Stewardship (AMS) resource section.

## 3. Discussion

### 3.1. CwPAMS App Metrics

Analysis of the CwPAMS App metrics revealed that the months with the highest downloads and page hits were September, October and November. The increase in September and October can be largely attributed to partnership project visits and antimicrobial stewardship interventions in all four countries. The spike in the month of November can most likely be linked to events during the World Antibiotic Awareness Week in all four countries as well as the app promotion by the Commonwealth Pharmacists Association during the World Antibiotic Awareness Week. Pharmacists accounted for the highest number of registered users and had more page hits and downloads than other health care professionals and workers. While this could mean that the app is more common among pharmacy teams, it calls for increased app promotion among doctors and other health professionals, who have also begun to use the app. The variations in the number of page hits and app downloads in each country can be explained by the number of partnerships in per country as Ghana and Uganda had the highest number of partnerships while Tanzania and Zambia had the lowest number of partnerships.

### 3.2. Cross-Sectional Survey

The use of smartphone mobile apps in healthcare delivery has gained acceptance over the years among patients and health professionals in sub-Saharan Africa and worldwide [19]. The CwPAMS App was developed by the Commonwealth Pharmacists Association to provide easy access to medicine management information for health professionals across Ghana, Tanzania, Uganda and Zambia. In addition to providing health professionals with relevant national and international guidelines, notable advantages of the app are its usability without internet access, a feature which suits low and middle-income countries, and its easy adaptability. Most recently, the app was updated to provide health care professionals across the commonwealth with links to relevant country-specific and international resources on COVID-19 from the World Health Organization (WHO), International Pharmaceutical Federation (FIP) and the Africa Centres for Disease Control and Prevention, among other relevant sources. The pilot study showed that more than 50% of patients were content with their health professional’s use of smartphone apps while attending to them. Age and education level had an impact on the patient’s acceptance of smartphone mobile technology as middle-aged patients had the least acceptance while the young and the most elderly had the greatest acceptance. Patients with tertiary education had the highest acceptance for these technologies while those with basic primary education had the least acceptance. These results correlate with a study carried out in 2014 on the acceptance and use of health technology by community-dwelling elders which revealed that income, education and age were found to significantly affect the acceptance of technology in healthcare. Patients with higher education and income used the internet at rates close to or exceeding the general population [25]. Another study also revealed that the acceptance of mobile phone technology among the older population was on the increase as they were found to constitute the fastest-growing group using the internet and computers [26]. Regarding patients’ preferences, our survey reveals that more patients preferred their health professionals using a computer/laptop to access information over a smartphone or reference book. This can be explained by the fact that the patients’ greatest concern was that smartphones could be a distraction to healthcare provision. This concern corroborates findings from a study by Wu et al. which revealed that on an average, physicians’ smartphones received 21.9 emails and 6.4 telephone calls, sent out 6.9 emails and initiated 8.3 telephone calls within 24 h. The study also revealed that 55.6% of 439 perfusionists admitted that they had used a cellular phone for purposes other than healthcare delivery while performing their duties [27]. On the contrary, a cross-sectional survey of adult patients in metropolitan academic and private dermatological clinics carried out in 2015 revealed that most patients (69.7%) considered personal smartphones an acceptable reference tool to provide information in patient care [28]. To access medical information more than once a day, health care workers mostly use mobile phones (60.5%) and printed posters (15.8%). These sources were also the most predominantly used daily (28.9% and 13.2%), respectively. This supports previous studies which have highlighted an increase in the use of smartphone mobile apps by health professionals [3,4,5]. Healthcare workers were also found to mostly consult internet search engines (50%), senior colleagues (36.8%) and pharmacists (31.6%) to access antibiotic prescribing information more than once a day. This demonstrates the need to involve these groups in promoting the app as they have a significant influence on antibiotic prescribing behaviours and healthcare workers’ decisions. Furthermore, healthcare professionals’ responses to the use of the CwPAMS App was found to correspond with results obtained from a similar study by Panesar et al. involving 146 healthcare professionals. Both studies show that the health professionals found apps useful and relevant to their patient population. They also agreed that apps encouraged them to challenge inappropriate prescribing [6]. The concern displayed by healthcare workers for the emergence of drug-resistant infections and the use of the standard treatment guidelines as seen in Table 6 was highly impressive. Healthcare workers also found the country-specific section of the CwPAMS App most useful. This correlates with the app metrics from all four countries which revealed that the National prescribing guidelines had the highest number of page hits from May 2019 to May 2020. The study highlights the need for more healthcare workers, especially doctors, to use the CwPAMS App as app metrics and the pilot cross-sectional survey both reveal that more nurses and pharmacists than doctors had used the app. There is also the need for more focused implementation as well as app promotion at all partnership sites and among all health professionals, especially doctors who are prescribers. Furthermore, there may be a need for subsequent studies to be carried out within the hospital when a higher number of healthcare professionals have used the app, in order to have a broader perspective from patients and health professionals. It would also be important to incorporate regular reminders about the app into the implementation strategy. A recently published study by Lester et al. [29] highlighted that implementing a locally appropriate, pragmatic antibiotic guideline through an app, supported by a simple educational strategy of weekly ‘reminders’, led to a significant reduction in third generation cephalosporin usage as well as an increase in the proportion of 48-h antibiotic reviews.

### 3.3. Strengths and Limitations

The CwPAMS Microguide antimicrobial prescribing app is the first of its kind to combine country-specific and international guidelines and information on antimicrobial prescribing for Ghana, Tanzania, Uganda and Zambia. Hence, based on our knowledge, this study on the development, implementation and use of the app in these four countries is novel. One of the limitations is the low sample size for the surveys, which was due to the time constraint in carrying out the survey, limited time spent by patients at the waiting room of a single hospital site and health care workers’ busy schedules. However, it is important to note that this section of the full study was intended to be a pilot in one setting and to provide initial descriptive findings. Extensive surveying across other sites would enable a test of significance and to confirm trends. In addition, the survey encompassed a wide range of health care workers, including doctors, pharmacists, nurses, midwives and other health care workers. Patients’ who participated where across a broad range with respect to age and education, providing a wide perspective. The response rate was greater for patients than health professionals, most likely because patients were available to fill questionnaires whilst in waiting rooms compared to health professionals. The proportion of healthcare workers groups that responded to the survey were not comparable. This is due to more nurses and other health care workers being available in the hospital compared to doctors and pharmacists. Though not all healthcare workers had used the app, there was an 85.7% response rate from those who had used the app to questions on the use of the App. Frequent updates and increased use of the app by health care workers highlight the need for further studies.

## 4. Materials and Methods

### 4.1. Development of the App

The CwPAMS App was developed by the Commonwealth Pharmacists Association using the MicroGuide platform (http://www.microguide.eu). The platform provides a cloud-based service that allows local pharmacists to develop, manage, update and publish clinical guidelines to various apps for any mobile operating system including iOS (Apple, Cupertino, CA, USA), Android (Google, Mountain View, CA, USA), Windows devices (Microsoft, Redmond, WA, USA) among other operating systems. It offers healthcare professionals offline access to clinical guidelines and content autonomously managed by pharmacy teams. It is also available online via https://viewer.microguide.global/CPA/CWPAMS. The CwPAMS App contains national and international guidelines listed into various sections including the WHO Essential Medicines List, surveillance tools, antimicrobial stewardship training, Infection Prevention and Control (IPC) resources, and country-specific Standard Treatment Guidelines. The App metrics and statistics were derived from routine data collection by Horizon Strategic Partners.

### 4.2. Study Site

The CwPAMS App was developed for use by 14 secondary care institutions that were part of the CwPAMS programme in four countries Ghana, Tanzania, Uganda and Zambia (S1–S3, Video S1). One of the hospitals in the partnership was used as the pilot study site. The hospital is a secondary health facility with a 100-bed capacity.

CwPAMS is a health partnership programme funded by the UK Department of Health and Social Care’s Fleming Fund to tackle antimicrobial resistance (AMR) globally. CwPAMS will support partnerships between the UK NHS and institutions in Ghana, Tanzania, Uganda and Zambia to work together on AMS initiatives. This aims to enhance implementation of protocols and evidenced based decision making to support antimicrobial prescribing, as well as capacity for antimicrobial surveillance. Further information about CwPAMS is available via https://commonwealthpharmacy.org/commonwealth-partnerships-for-antimicrobial-stewardship/. CwPAMS is being run by the Commonwealth Pharmacists Association (CPA) and Tropical Health Education Trust (THET).

### 4.3. Study Design

The CwPAMS App metrics were obtained from data collected by the Horizon Strategic Partners. These assessed the frequency of page hits, guide opens and the number of registered users and downloads. The pilot study was a cross-sectional survey with patients and healthcare workers in one of the hospital sites, six months after the introduction of the App using questionnaires adapted from Panesar et al. [6]. Patients’ questionnaires comprised of four sections with eight questions using a Likert scale and multiple-choice questions. The first section comprised of demographics including age, gender, highest education qualification and occupation. The second section assessed patients’ attitudes to health professionals’ use of smart phone mobile apps in healthcare delivery. The third section was designed to obtain patients’ concerns about the use of these smart phone apps, while the last section requested patients’ preferences for health professionals reference ranging from a smart phone mobile app to a tablet, computer/laptop and a reference book. The health care workers’ questionnaires comprised of nine sections with 15 questions designed as a Likert scale and open-ended questions. The first section obtained healthcare workers’ demographics including country, specialty, year of graduation, grade, type of institution and profession and role. The eight sections following comprised of health professionals’ attitudes to the use of the CwPAMS App and current practices.

### 4.4. Sample Size Determination/Sample Size and Sampling Technique

A convenience sample size determination of maximum 100 each was used for the cross-sectional study.

### 4.5. Procedure for Data Collection

#### 4.5.1. CwPAMS App metrics

App metrics for user engagement evaluating the number of registered users, downloads, guide opens and page hits for various sections of the App from April 2019 to May 2020 were obtained through the MicroGuide platform. (http://www.microguide.eu).

#### 4.5.2. Pilot Cross-Sectional Survey

Health Professionals Survey: Questionnaires were distributed among healthcare workers comprising of doctors, pharmacists, nurses and other healthcare workers at various points of care in the hospital including consulting rooms, nurses’ station, pharmacy sections and wards. A total of 100 questionnaires were distributed to health professionals with 38 returned questionnaires completed anonymously.

Patients Survey: Patients’ questionnaires were distributed to patients in the waiting room within the consulting area. Patients’ questionnaires comprised of demographic data and questions regarding attitude to the use of smartphone apps among health professionals over a one-week period. Patients’ consent was sought for before administration of the questionnaires. A total of 93 questionnaires were distributed to patients based on patients available in hospital during the study period. All 47 questionnaires (S4: Questionnaires) were completed anonymously with no personally identifiable information documented.

#### 4.5.3. Study Approval

Study was conducted under service improvement as part of the CwPAMS project therefore no ethical approval was required but the Ghana Health Service and the Ghana AMR Platform were made aware of the pilot project.

### 4.6. Procedure for Data Analysis

Microsoft Excel 2013 was used to analyse the data obtained from the pilot study using descriptive statistics.

## 5. Conclusions

Our study provides insight into the overall perception of the use of mobile apps as a means to improve antimicrobial stewardship, demonstrating general acceptance among patients and healthcare professionals. In general, the patients and healthcare workers surveyed had a positive attitude following the introduction of the CwPAMS App as a fundamental resource for accessing information on antimicrobial prescribing. Hence, increased and more comprehensive use of all sections of the App could contribute to improved antimicrobial stewardship practices among healthcare workers and increased acceptance of the use of smartphone apps among patients. App downloads and utilization were found to be highest during partnership visits and App promotion, highlighting the need for more focused implementation and promotion of the App among all health professionals, especially doctors. Further studies will be useful in evaluating the impact of the App on antimicrobial prescribing as well as guide future Antimicrobial Stewardship interventions.

## Figures and Tables

**Table 1:** Frequency of monthly downloads, guide opens and page hits for Ghana, Tanzania, Uganda and Zambia.

Commonwealth Partnerships for Antimicrobial Stewardship (CwPAMS) App Metrics															
	2019									2020					Total (%)
Months	Apr	May	Jun	Jul	Aug	Sep	Oct	Nov	Dec	Jan	Feb	Mar	Apr	May	
**Downloads (n)**															
		12	20	75	21	67	66	124	27	28	27	9	9	40	100
**Guide Opens (n)**															
	35	36	26	354	237	453	375	457	147	141	130	127	122	155	100
**National Guideline Page Hits by Country (n)**															
**Ghana**		40	17	569	176	399	827	438	192	141	375	243	126	46	50.3
**Tanzania**		3	11	101	21	20	53	307	73	25	27	187	73	22	13
**Uganda**		21	81	196	223	663	322	368	64	101	96	10	13	45	31
**Zambia**		2	-	15	4	8	9	74	4	3	0	4	10	4	1.9
**Nil**		17	7	65	27	19	23	39	10	24	3	19	15	6	3.8
**Individual Page Hits by App Sections (n)**															
**About the CwPAMS** **App**		13	9	57	39	44	31	71	11	13	8	19	16	37	3.4
**Userguide**		3	4	58	31	38	29	58	17	17	8	9	22	15	2.9
**National Prescribing Guidelines**		83	116	946	451	1109	1234	1226	343	294	501	463	237	119	66.1
**Access, Watch, Reserve (AWaRE)—WHO** **Essential** **Medicines List**		132	17	140	212	184	54	202	45	39	18	40	49	42	11.0
**Antimicrobial Stewardship (AMS) Tools**		21	3	84	143	89	40	72	17	36	10	25	18	27	5.5
**Training on AMS**		26	4	105	105	66	46	51	7	33	10	20	10	4	4.5
**Infection Prevention and Control**		6	5	91	61	42	24	70	24	17	7	16	23	18	3.7
**Antimicrobial Use Surveillance**		11	2	44	43	39	15	26	8	14	4	11	16	3	2.2
**Updates on Antimicrobial Resistance (AMR)—Coming Soon**		4	1	8	7	7	9	11	0	11	1	0	8	0	0.7

**Table 2:** Frequency of registered users, downloads and guide opens by profession.

Profession	CwPAMS App Metrics (By Profession)		
	Registered Users	Downloads	Guide Opens
Dentistry	1	2	2
Physiotherapy	1	0	0
Clinical science	3	0	0
Healthcare management	6	2	1
Paramedic	6	1	1
Biomedical scientist	15	3	11
Physician Associate	15	1	12
Medicine	84	57	80
Nursing	103	29	36
Pharmacy	258	159	274
Other	13	2	3

**Table 3:** Demographics of patients and healthcare professionals.

Patients							
Highest Level of Education Obtained							
Basic Primary Education		Secondary Education		Tertiary Education		No data provided	
4		23		19		1	
**Age**							
<18	18–25	26–35	36–45	46–55	56–67	68 above	Nil
1	25	7	1	3	5	3	2
**Health Professionals**							
Profession							
Doctor		Pharmacist		Nurse		Others	
4		6		18		10	

**Table 4:** Patients’ responses to the use of smart phone mobile apps in healthcare delivery—5-point Likert scale from Strongly disagree (1) to Strongly agree (5) (n = 47).

Questions/Comments	Score (%)				
	1	2	3	4	5
**What do you feel about the following?**					
I am pleased with my doctor assessing a smart phone app while attending to me	21.3	36.2	23.4	6.4	12.8
Using smart phone apps will increase the quality of healthcare offered by my doctor	25.5	31.9	19.1	17.0	6.4
The use of smart phone apps quickens access to healthcare	17.0	40.4	25.5	12.8	4.3
The use of smart phone apps increases quality of healthcare delivery	14.9	36.2	36.2	6.4	6.4
**Do you have any reservations/concerns with a doctor’s use of a mobile app while attending to you?** **If yes, what are your concerns?**					
The doctor may not be competent enough	4.3	17.0	8.5	10.6	12.8
It is a distraction to healthcare provision	4.3	25.5	12.8	10.6	2.1
My data may not be protected/secured	6.4	21.3	10.6	8.5	4.3
Mobile devices may not be technically reliable enough	6.4	21.3	10.6	17.0	0
The use of smart phones/mobile apps may be complicated when it comes to healthcare	4.3	17.0	12.8	17.0	2.1

**Table 5:** Use of the Commonwealth Partnerships for Antimicrobial Stewardship (CwPAMS) App and other sources of information by healthcare workers (n = 38).

Frequency of Accessing Medical Information (%)						
Device	Daily	More than once a day	Weekly	Monthly	Never	Nil
Mobile phone	28.9	60.5	5.3	2.6	0	2.6
Tablet	7.9	13.2	5.3	0	47.4	26.3
Computer	7.9	10.5	5.3	26.3	21.0	28.9
Pocket book	10.5	2.6	13.2	10.5	28.9	34.2
Printed posters	13.2	15.8	7.9	13.2	26.3	23.7
Others	0	5.3	0	0	5.3	89.5
**Frequency of Accessing Antimicrobial Prescribing Information (%)**						
CwPAMS App	2.6	5.3	7.9	2.6	63.2	18.4
Printed copy of standard treatment guidelines	26.3	21.0	0	21.0	13.2	18.4
British National Formulary (BNF)/National guidelines	28.9	23.7	10.5	15.8	10.5	10.5
Microbiology/Infectious disease advice	13.2	10.5	15.8	7.9	31.6	21.0
Pharmacists	18.4	31.6	7.9	15.8	13.2	13.2
Senior colleagues	26.3	36.8	7.9	13.2	2.6	13.2
Other junior doctors	26.3	26.3	2.6	10.5	18.4	15.8
Internet search engines (e.g., Google)	18.4	50.0	13.2	2.6	2.6	13.2
Others	0	0	0	0	2.6	97.4

**Table 6:** Assessment of Standard treatment guidelines and drug resistant Infections on a 5-point Likert scale from Strongly Agree (1) to Strongly Disagree (5), median response category for each question marked in bold.

Questions/Comments	Score				
	1	2	3	4	5
Assessment of standard treatment guidelines and attitude to Antimicrobial Resistance (%) (n = 38)					
Standard antimicrobial treatment guidelines are easy to access	15.8	**63.2**	10.5	5.3	5.3
My seniors’ preferences guide antimicrobial prescribing more than standard treatment guidelines	7.9	36.8	**26.4**	15.8	2.6
Standard antimicrobial treatment guidelines don’t apply to my patients	7.9	5.3	13.2	**42.1**	26.4
I prefer to use non-standard treatment guidelines to guide my antimicrobial prescribing	5.3	13.2	7.9	**36.8**	28.9
I am concerned about the emergence of drug-resistant infections	**50**	44.7	0	0	0

## Supplementary Materials

The following are available online at https://www.mdpi.com/2079-6382/9/9/555/s1: S1: Launch Communications presentation, S2: AMS App–Commonwealth Pharmacists Association (CPA) Press Release https://commonwealthpharmacy.org/ams-app-cpa-press-release/, Video S1: Commonwealth Partnerships for Antimicrobial Stewardship App https://www.youtube.com/watch?v=MJ7fa_aLgCI, S3: App Launch Posters, S4: Questionnaires Healthcare workers and patients.
