# Supplementary material for: Improving Access to Antimicrobial Prescribing Guidelines in 4 African Countries: Development and Pilot Implementation of an App and Cross-Sectional Assessment of Attitudes and Behaviour Survey of Healthcare Workers and Patients
Source: Antibiotics (Basel). 2020 Aug 29;9(9):555. doi: 10.3390/antibiotics9090555 (PMC7558264; doi:10.3390/antibiotics9090555)
Supplement: Supplementary file 1 [file antibiotics-09-00555-s001.zip › S1_ 'Communications presentation on the CwPAMS App (1).pptx'.pdf]

The Fleming Fund

UKaid from the British people

THET PARTNERSHIPS FOR GLOBAL HEALTH

COMMONWEALTH PHARMACISTS ASSOCIATION

Introducing…..

The new CwPAMS prescribing app

Medicines management information at your fingertips!

Tweet us!
#CwPAMS

The CwPAMS Microguide app

The CwPAMS app has been developed to provide easy access to information that is vital to use antimicrobials approp[illegible]

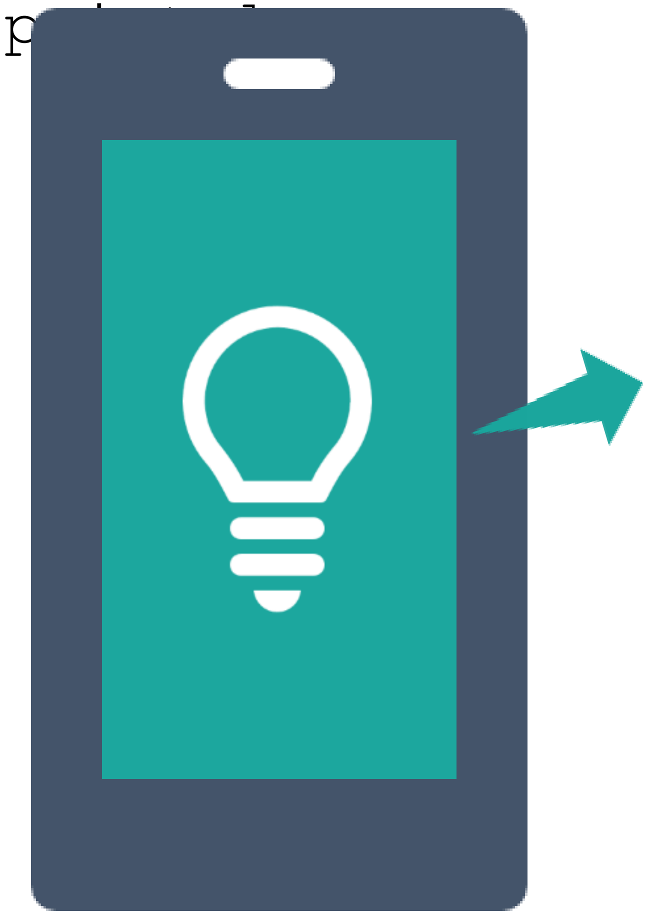

It has many benefits…

- Easy download and Offline mode for poor internet connectivity
- All the information you need for antimicrobial prescribing and more at your fingertips
- You can tailor 'favourite' pages for quick access
- COMPLETELY FREE

The Fleming Fund
UKaid from the British people
THET PARTNERSHIPS FOR GLOBAL HEALTH
COMMONWEALTH PHARMACISTS ASSOCIATION

Medicines information management

- The app provides World Health Organisation (WHO) and National medicines information

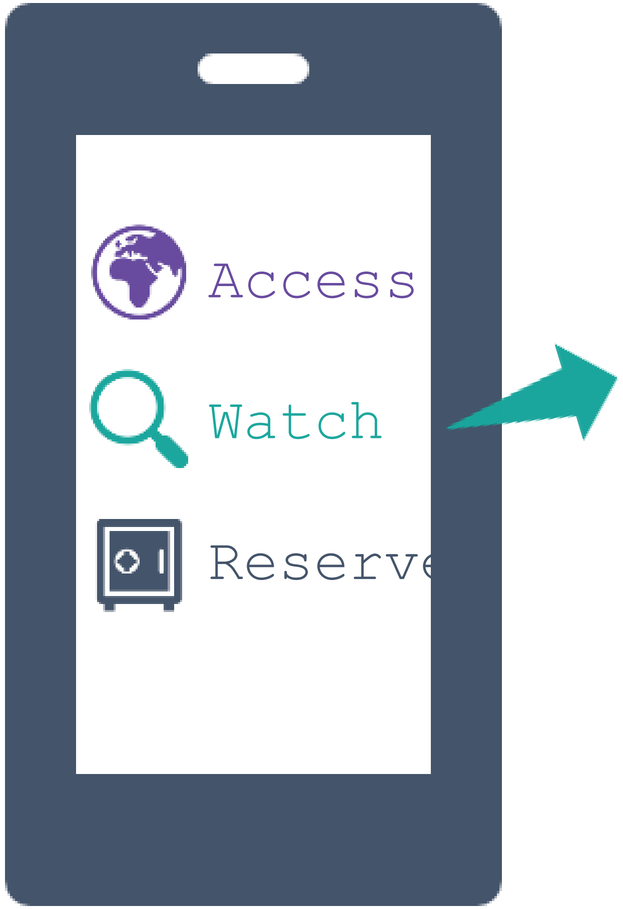

WHO essential medicines lists on antimicrobials

- Antibiotics (Access, Watch, Reserve – AWaRe)
- AntiTB, antifungal and antimalarial medicines

National standard treatment guidelines on antimicrobials

The Fleming Fund
UKaid from the British people
THET PARTNERSHIPS FOR GLOBAL HEALTH
COMMONWEALTH PHARMACISTS ASSOCIATION

Antimicrobial stewardship tools

- Tools to support antimicrobial stewardship such as

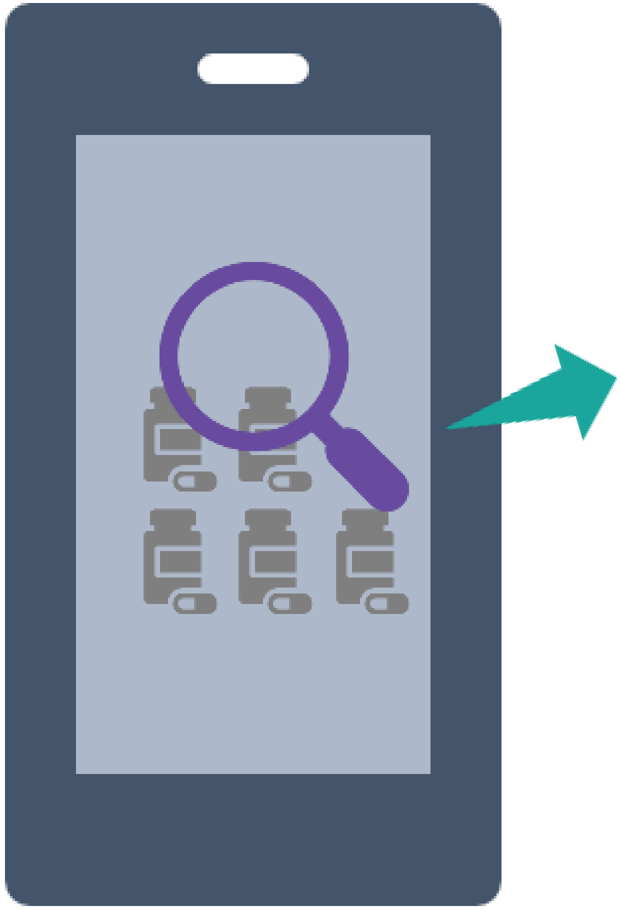

- AMS Checklist tool
- AMS training resources
- Infection prevention and control (IPC) and water, sanitation and hygiene (WASH) training and assessment tools
- Global PPS tools for surveillance of antimicrobials

The Fleming Fund
UKaid from the British people
THET PARTNERSHIPS FOR GLOBAL HEALTH
COMMONWEALTH PHARMACISTS ASSOCIATION

How to download – three easy steps

1. In your app store download the Microguide app
2. Set up a user and select Commonwealth Pharmacists' Association as institution
3. Select the CwPAMS guide and download (It will automatically update as new versions come available)

DESKTOP MODE: Available on computers via the link:

https://viewer.microguide.global/CPA/CWPAMS

The Fleming Fund UKaid from the British people THET PARTNERSHIPS FOR GLOBAL HEALTH COMMONWEALTH PHARMACISTS ASSOCIATION

CwPAMS App

- About the CwPAMS app
- Userguide
- AWaRE - WHO Essential Medicines List of Antibacterials
- National prescribing guidelines
- Antimicrobial stewardship tools
- Antimicrobial use surveillance
- Training on antimicrobial stewardship
- Updates on antimicrobial resistance - coming soon

National prescribing guidelines

- Ghana antimicrobial prescribing guidelines
- Tanzania antimicrobial prescribing guidelines
- Uganda antimicrobial prescribing guidelines
- Zambia antimicrobial prescribing guidelines

The Fleming Fund
UKaid from the British people
THET PARTNERSHIPS FOR GLOBAL HEALTH
COMMONWEALTH PHARMACISTS ASSOCIATION

Ghana antimicrobial prescribing guidelines

- About
- Disorders of the gastrointestinal tract
- Disorders of the liver
- Nutritional disorders
- Hematological disorders
- Immunisable diseases
- Problems of the new born (neonate)

Immunisable diseases

- Pertussis
- Tetanus
- Diphtheria
- Haemophilus Influenzae type b Disease
- Pneumococcal Disease
- Hepatitis

The Fleming Fund
UKaid from the British people
THET PARTNERSHIPS FOR GLOBAL HEALTH
COMMONWEALTH PHARMACISTS ASSOCIATION

Eradication of bacteria in a patient diagnosed to have tetanus

Eradication of bacteria in a patient diagnosed to have tetanus
1st Line Treatment
Evidence Rating: [A]
**Metronidazole, IV,**
Adults
500 mg 6 hourly for 7-10 days
Children
> 1 month 7.5 mg/kg 8 hourly for 7-10 day
Neonates
> 7 days; 7.5 mg/kg 12 hourly
< 7 days; 7.5 mg/kg 48 hourly
2nd Line Treatment
Evidence Rating: [B]
**Benzylpenicillin, IV,**
Adults
50,000 units/kg stat, then 4 MU 6 hourly for 5 days
Children
50,000 units/kg 6 hourly for 5 days
Neonates
250,000 units 6 hourly for 7 days
And
**Gentamicin, IV, (neonates only), 4 mg/kg 24 hourly**

The Fleming Fund
UKaid from the British people
THET PARTNERSHIPS FOR GLOBAL HEALTH
COMMONWEALTH PHARMACISTS ASSOCIATION
