# Supplementary material for: Improving Access to Antimicrobial Prescribing Guidelines in 4 African Countries: Development and Pilot Implementation of an App and Cross-Sectional Assessment of Attitudes and Behaviour Survey of Healthcare Workers and Patients
Source: Antibiotics (Basel). 2020 Aug 29;9(9):555. doi: 10.3390/antibiotics9090555 (PMC7558264; doi:10.3390/antibiotics9090555)
Supplement: Supplementary file 1 [file antibiotics-09-00555-s001.zip › S3_AppLaunch - generic.pdf]

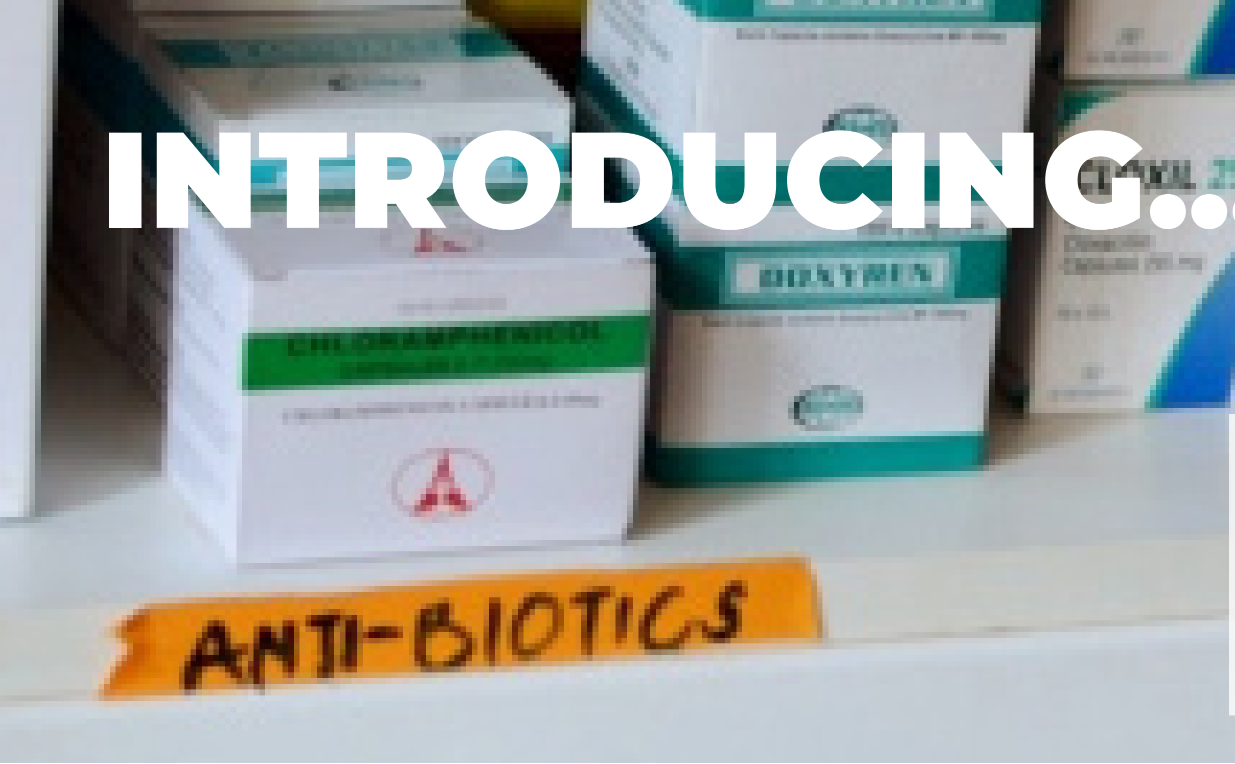

INTRODUCING…

COMMONWEALTH **PHARMACISTS** ASSOCIATION

The new CwPAMS Infection App

Download MicroGuide from your App store and select Commonwealth Pharmacists Association (CPA) as your institution.

•Search for **MicroGuide** in app store - download
•Click on the following: get guide - select medical organisation - click inside circle **(Commonwealth Pharmacists Association).** Click CwPAMS app, finally, on top left click get selected guide

…Instant access to up-to-date national guidelines at the point of care.

✓Improves patient care
✓Saves time
✓Increases diagnostic accuracy
✓Helps regulate prescribing practices
✓Enhances physician efficiency.

UKaid from the British people

The Fleming Fund

COMMONWEALTH PHARMACISTS ASSOCIATION

THET PARTNERSHIPS FOR GLOBAL HEALTH

BECOME AN ANTIBIOTIC GUARDIAN
