# Supplementary material for: Improving Access to Antimicrobial Prescribing Guidelines in 4 African Countries: Development and Pilot Implementation of an App and Cross-Sectional Assessment of Attitudes and Behaviour Survey of Healthcare Workers and Patients
Source: Antibiotics (Basel). 2020 Aug 29;9(9):555. doi: 10.3390/antibiotics9090555 (PMC7558264; doi:10.3390/antibiotics9090555)
Supplement: Supplementary file 1 [file antibiotics-09-00555-s001.zip › S3_AppLaunch - patient poster.pdf]

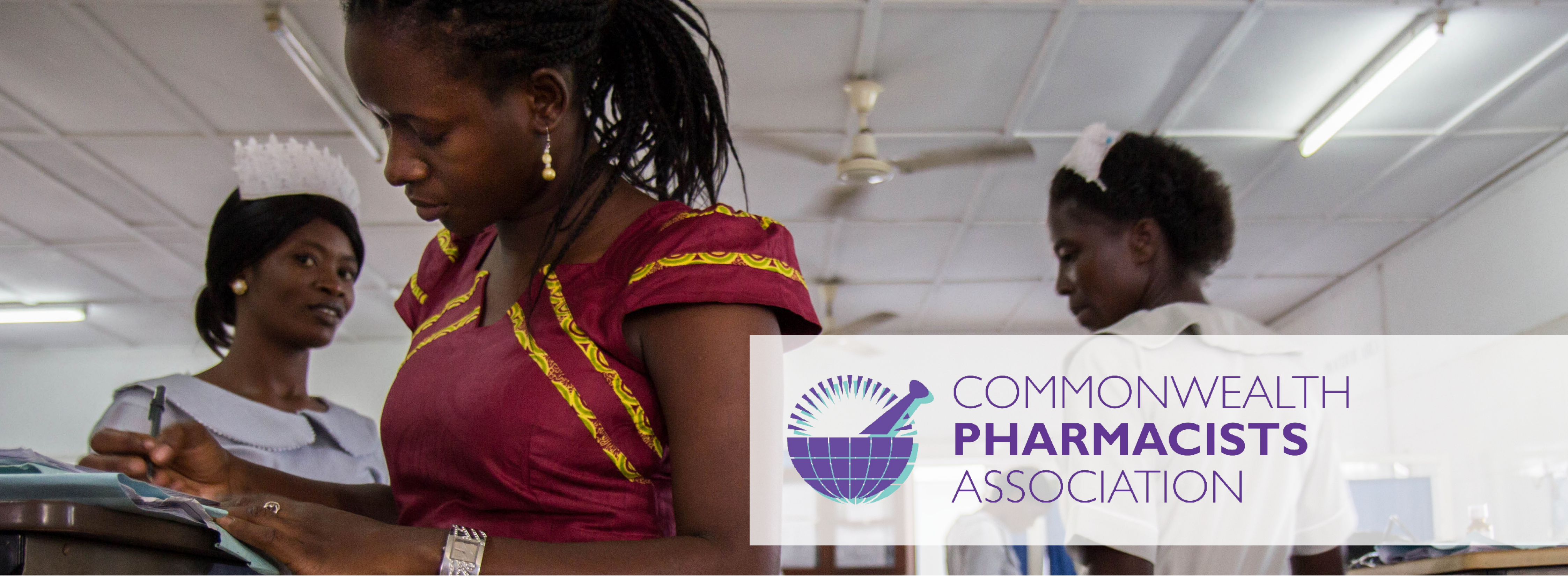

COMMONWEALTH **PHARMACISTS** ASSOCIATION

DID YOU KNOW...

YOUR DOCTOR, NURSE OR PHARMACIST IS ENSURING THE BEST DRUG AND MOST UP TO DATE CARE RECOMMENDED FOR YOU WHEN THEY USE THE MEDICINES INFORMATION APP.

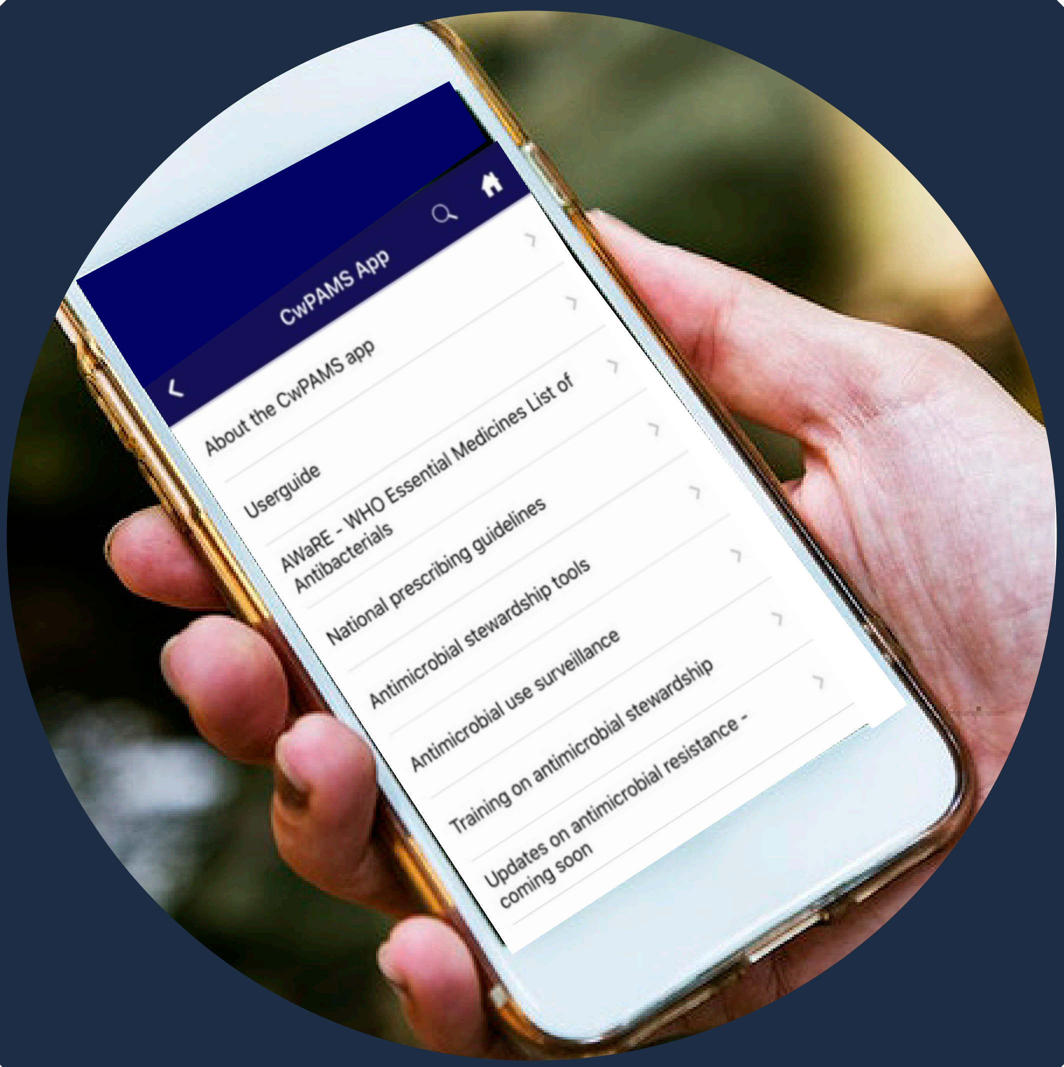

UKaid from the British people
The Fleming Fund
COMMONWEALTH PHARMACISTS ASSOCIATION
THET PARTNERSHIPS FOR GLOBAL HEALTH
BECOME AN ANTIBIOTIC GUARDIAN
Keep Antibiotics Working
