# Supplementary material for: Improving Access to Antimicrobial Prescribing Guidelines in 4 African Countries: Development and Pilot Implementation of an App and Cross-Sectional Assessment of Attitudes and Behaviour Survey of Healthcare Workers and Patients
Source: Antibiotics (Basel). 2020 Aug 29;9(9):555. doi: 10.3390/antibiotics9090555 (PMC7558264; doi:10.3390/antibiotics9090555)
Supplement: Supplementary file 1 [file antibiotics-09-00555-s001.zip › S3_AppLaunch_1_Ghana.pdf]

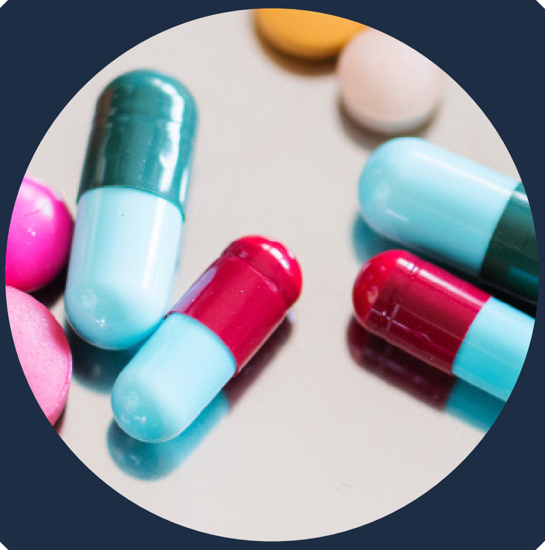

GHANA

ANTIMICROBIAL PRESCRIBING GUIDELINES AT YOUR FINGERTIPS!

The CwPAMS app provides easy access to medicines management information to support appropriate antimicrobial stewardship with these essential features:

CwPAMS App

- About the CwPAMS app
- Userguide
- AWaRE - WHO Essential Medicines List of Antibacterials
- National prescribing guidelines
- Antimicrobial stewardship tools
- Antimicrobial use surveillance
- Training on antimicrobial stewardship
- Updates on antimicrobial resistance - coming soon
- Infection prevention control

- How to use the guidelines
- WHO Essential Medicines List of Antibiotics
- WHO Essential Medicines List of Antifungals
- WHO Essential List Antituberculosis Medicines
- WHO Essential List of Antimalarials

- Ghana antimicrobial prescribing guidelines 2017
- Tanzania antimicrobial prescribing guidelines 2017
- Uganda antimicrobial prescribing guidelines 2016
- Zambia antimicrobial prescribing guidelines 2013

- WHO IPC assessment tools
- Links to training resources
- Guidance documents on IPC

How to download:

1. Search for MicroGuide in app store - download
2. Click on the following: get guide - select medical organisation - click inside circle (Commonwealth Pharmacists Association).
3. Click CwPAMS app, finally, on top left click get selected guide.

UKaid from the British people | The Fleming Fund | COMMONWEALTH PHARMACISTS ASSOCIATION | THET PARTNERSHIPS FOR GLOBAL HEALTH | BECOME AN ANTIBIOTIC GUARDIAN
