# Supplementary material for: Improving Access to Antimicrobial Prescribing Guidelines in 4 African Countries: Development and Pilot Implementation of an App and Cross-Sectional Assessment of Attitudes and Behaviour Survey of Healthcare Workers and Patients
Source: Antibiotics (Basel). 2020 Aug 29;9(9):555. doi: 10.3390/antibiotics9090555 (PMC7558264; doi:10.3390/antibiotics9090555)
Supplement: Supplementary file 1 [file antibiotics-09-00555-s001.zip › S4_CwPAMS App-Patient questionnaire.pdf]

Survey on antimicrobial prescribing app in Ghana

Age:

☐ 18-25yrs ☐ 26-35yrs ☐ 36-45yrs

☐ 46-55yrs ☐ 56-67yrs ☐ 68yrs and above

Gender

☐ Male ☐ Female

Highest level of education obtained

☐ Basic primary education

☐ Secondary education

☐ Tertiary education (Higher than secondary)

Occupation

……………………………

Has a physician ever used a smart phone app while attending to you?

Yes ☐ No ☐

What do you feel about the following?

	**Strongly agree**	**Agree**	**Neutral**	**Disagree**	**Strongly disagree**
I am pleased with my doctor accessing a smart phone mobile app while attending to me.					
Using smart phone apps will increase the quality of healthcare offered by my doctor.					
The use of smart phone apps quickens access to health care.					
The use of smart phone apps increases quality of health care delivery.					

Do you have any reservations/concerns with a doctor's use of a mobile app while attending to you?

Yes ☐ No ☐

If yes, what are your concerns?

	Strongly agree	Agree	Neutral	Disagree	Strongly disagree
The doctor may not be competent enough.					
It is a distraction to healthcare provision.					
My data may not be protected /secured.					
Mobile devices may not be technically reliable enough.					
The use of smart phones /mobile apps might be complicated when it comes to healthcare.					

Any additional concerns? Kindly state them

……

……

……

……

Which of the following would you prefer to have your physician use?

☐ A smart mobile phone app ☐ A tablet

☐ A computer/laptop ☐ A reference book
