# Supplementary material for: Improving Access to Antimicrobial Prescribing Guidelines in 4 African Countries: Development and Pilot Implementation of an App and Cross-Sectional Assessment of Attitudes and Behaviour Survey of Healthcare Workers and Patients
Source: Antibiotics (Basel). 2020 Aug 29;9(9):555. doi: 10.3390/antibiotics9090555 (PMC7558264; doi:10.3390/antibiotics9090555)
Supplement: Supplementary file 1 [file antibiotics-09-00555-s001.zip › S4_CwPAMS App_LAUNCH-HCW Questionnaire.pdf]

Survey on antimicrobial prescribing app in Ghana

1) Background information

Country…………………………………………..   Grade ………………………………………….

Specialty…………………………………………..   Type of institution…………………………………………..

Year of graduation ……………………………………   Profession (circle one): Doctor/Nurse/Pharmacist/Other

Which do you do most frequently in your role (circle one): prescribe / administer /dispense

2) How frequently do you access <u>ANY FORM OF MEDICAL INFORMATION</u> using the following:

	More than once a day	Daily	Weekly	Monthly	Never
A mobile phone	☐	☐	☐	☐	☐
A tablet	☐	☐	☐	☐	☐
A computer	☐	☐	☐	☐	☐
A pocket book	☐	☐	☐	☐	☐
Printed posters	☐	☐	☐	☐	☐
Other (please state) …………………………………………….	☐	☐	☐	☐	☐

	More than once a day	Daily	Weekly	Monthly	Never
**3) How frequently do you prescribe/administer or dispense <u>antimicrobials</u>?**	☐	☐	☐	☐	☐

4) Please tick the most appropriate answers to the statements below. <u>(pls tick one option per line)</u>

	Strongly disagree	Disagree	Neither agree nor disagree	Agree	Strongly agree
Standard antimicrobial treatment guidelines are easy to access	☐	☐	☐	☐	☐
My seniors' preferences guide antimicrobial prescribing more than standard treatment guidelines	☐	☐	☐	☐	☐
Standard antimicrobial treatment guidelines don't apply to my patients	☐	☐	☐	☐	☐
I prefer to use non-standard treatment guidelines to guide my antimicrobial prescribing	☐	☐	☐	☐	☐
I am concerned about the emergence of drug-resistant infections	☐	☐	☐	☐	☐

5) Do you carry a smartphone with you at work?

Yes No

6) Which operating system does it run on?

iPhone Android Windows Phone Blackberry Other (please state) ……………………………………..

7) How frequently did/do you access the following sources of information on <u>ANTIMICROBIAL PRESCRIBING</u>? <u>(Tick one option per line)</u>

	More than once a day	Daily	Weekly	Monthly	Never
CwPAMS App	☐	☐	☐	☐	☐
Printed copy of Standard treatment guidelines	☐	☐	☐	☐	☐
BNF / National guidelines	☐	☐	☐	☐	☐
Microbiology / Infectious Diseases advice	☐	☐	☐	☐	☐
Pharmacists	☐	☐	☐	☐	☐
Senior colleagues	☐	☐	☐	☐	☐
Other junior doctors	☐	☐	☐	☐	☐
Internet search engines (e.g. Google)	☐	☐	☐	☐	☐
Other (please state) ……………………………………………………………….	☐	☐	☐	☐	☐

8a) Are you aware that the infection treatment from the Ghana Standard Treatment guidelines is available as an Smartphone App? (circle one) Yes No

8b) Have you downloaded the app? (circle one) Yes No

9) Only answer this question <u>IF you have downloaded the app</u> - How frequently do you now access the following sources of information on <u>ANTIMICROBIAL PRESCRIBING</u>? <u>(Tick one option per line)</u>

	More than once a day	Daily	Weekly	Monthly	Never
CwPAMS App	☐	☐	☐	☐	☐
Printed copy of standard treatment guidelines	☐	☐	☐	☐	☐
BNF / National guidelines	☐	☐	☐	☐	☐
Microbiology / Infectious Diseases advice	☐	☐	☐	☐	☐
Pharmacists	☐	☐	☐	☐	☐
Senior colleagues	☐	☐	☐	☐	☐
Other junior doctors	☐	☐	☐	☐	☐

Internet search engines (e.g. Google)	☐	☐	☐	☐	☐
Other (please state) …………………………………………………………….	☐	☐	☐	☐	☐

10) If you have downloaded the app but not currently using regularly please share what the challenges are:

____

____

11) Please tick the most appropriate answers to the statements below about <u>THE CwPAMS SMART PHONE APP (Tick one option per line)</u>

	Strongly disagree	Disagree	Neither agree nor disagree	Agree	Strongly agree
I have found the app useful	☐	☐	☐	☐	☐
The app is easy to navigate	☐	☐	☐	☐	☐
The content of the app is relevant to my - patient population	☐	☐	☐	☐	☐
The app is the best way to access standard antimicrobial treatment guidelines	☐	☐	☐	☐	☐
I would like to see all standard antimicrobial treatment guidelines on the app	☐	☐	☐	☐	☐
The app encourages me to document the **indication** for antimicrobials on the drug chart	☐	☐	☐	☐	☐
The app encourages me to document the **duration** for antimicrobials on the drug chart	☐	☐	☐	☐	☐
The app encourages me to challenge inappropriate prescribing by others	☐	☐	☐	☐	☐
I feel comfortable using a smart phone on a ward round	☐	☐	☐	☐	☐
I feel comfortable accessing the app at the patient's bedside	☐	☐	☐	☐	☐
The app has increased my awareness of antimicrobial stewardship	☐	☐	☐	☐	☐

12) Please, rate how useful you found each of the following sections in the app <u>(Tick one option per line)</u>

	Very useful	Useful	Not useful	Didn't use
The WHO Essential Medicines list section	☐	☐	☐	☐
The country specific standard treatment guidelines section	☐	☐	☐	☐
The AMS resource section	☐	☐	☐	☐
The Global PPS tools section	☐	☐	☐	☐
The IPC tools section	☐	☐	☐	☐

13) Do you have any suggestions for improving the content of the CwPAMS smart phone app? ....

14) Please detail any other comments you have about the CwPAMS antimicrobial smart phone app ....

15) How many other medical apps do you use on a regular basis? Please state their names. ....

Please use the section below to share any other feedback or comments:
